# A model for predicting the phenology of *Philaenus spumarius*

**DOI:** 10.1038/s41598-024-58798-x

**Published:** 2024-04-07

**Authors:** Gianni Gilioli, Anna Simonetto, Igor Daniel Weber, Paola Gervasio, Giorgio Sperandio, Domenico Bosco, Nicola Bodino, Crescenza Dongiovanni, Michele Di Carolo, Vincenzo Cavalieri, Maria Saponari, Donato Boscia

**Affiliations:** 1 DICATAM, University of Brescia, Via Branze 43, 25123 Brescia, Italy; 2 Marche Polytechnic University, D3A, Via Brecce Bianche 10, 60131 Ancona, Marche Italy; 3 DISAFA, University of Turin, Largo Paolo Braccini, 10095 Grugliasco, TO Italy; 4 Centro di Ricerca, Sperimentazione e Formazione in Agricoltura Basile Caramia, Locorotondo, Italy; 5 Consiglio Nazionale delle Ricerche, Istituto per la Protezione Sostenibile delle Piante, Sede Secondaria di Bari, Bari, Italy

**Keywords:** Developmental biology, Ecology

## Abstract

The design and implementation of *Philaenus spumarius* control strategies can take advantage of properly calibrated models describing and predicting the phenology of vector populations in agroecosystems. We developed a temperature-driven physiological-based model based on the system of Kolmogorov partial differential equations to predict the phenological dynamics of *P. spumarius*. The model considers the initial physiological age distribution of eggs, the diapause termination process, and the development rate functions of post-diapausing eggs and nymphal stages, estimated from data collected in laboratory experiments and field surveys in Italy. The temperature threshold and cumulative degree days for egg diapause termination were estimated as 6.5 °C and 120 DD, respectively. Preimaginal development rate functions exhibited lower thresholds ranging between 2.1 and 5.0 °C, optimal temperatures between 26.6 and 28.3 °C, and upper threshold between 33.0 and 35 °C. The model correctly simulates the emergence of the 3rd, 4th, and 5th nymphal instars, key stages to target monitoring actions and control measures against *P. spumarius*. Precision in simulating the phenology of the 1st and 2nd nymphal stages was less satisfactory. The model is a useful rational decision tool to support scheduling monitoring and control actions against the late and most important nymphal stages of *P. spumarius*.

## Introduction

The meadow spittlebug, *Philaenus spumarius* L. (1758) (Hemiptera: Aphrophoridae), is a xylem sap-feeder widely spread in the world and is considered the main vector of *Xylella fastidiosa* Wells (1987) (Proteobacteria: Xanthomonadaceae) (*Xf*) in Europe^1,2^. *Xf* is a xylem-limited Gram-negative bacterium responsible for severe diseases in ornamental and economically valuable crops, including Pierce’s disease of grapevine, citrus variegated chlorosis, almond leaf scorch, and olive quick decline syndrome. *Xf* is listed as a priority pest in Europe given the serious threat posed by the species from the economic and environmental point of view^2,3^. The importance of *P. spumarius* as a vector of *Xf* in Europe is mainly associated with its higher abundance in the ongoing *Xf* outbreak areas compared to other xylem sap-feeding species, and its transmission efficiency exceeds that of other *Xf*-vectors present in Europe^4–6^.

The meadow spittlebug has a one-year life cycle. Overwintering eggs leave diapause as the temperatures increase in late winter/early spring, when they complete their development and hatch^1,7,8^. Cold temperatures may contribute to accelerate diapause termination once the eggs are exposed to warm temperatures, although eggs can leave diapause without being exposed to cold^7–9^. *P. spumarius* has five nymphal instars which develop mainly on spontaneous herbaceous cover, where it produces a spittle for shelter^1^. In the Mediterranean conditions, the first adults emerge in the second half of April and May and move onto woody plants (e.g. shrubs, olive and wild trees) in late spring/early summer, when herbaceous covers become less suitable due to the dry climatic conditions^4,10^. Newly emerged adult females undergo an ovarian parapause that delays ovarian maturation and oviposition during long daylight periods^8,9^. Adults return to herbaceous cover in late summer/early autumn for oviposition, when the overall adult population decreases until late autumn. Generally, adults do not overwinter^1,10^.

Adult vectors acquire *Xf* bacterial cells while feeding from infected xylem vessels. Infected vectors can immediately transmit the bacterium to other plants with no latency on subsequent feedings^11^. Juveniles of *P. spumarius* do not play an active role in the *Xf* transmission given their limited dispersal capacity and host-plant range, restricted to herbaceous species^4,10^. However, the timely control of the vector, especially the juveniles and the early-emerged adults, in the outbreak area is recommended to reduce the incidence and spread of *Xf*^2,5^.

The development of accurate phenological models describing the phenology of *P. spumarius* populations in agroecosystems is fundamental to support rational decision-making for the efficient management of the vector population^12^. These quantitative tools ensure that control measures can be applied timely, targeting the most vulnerable stages of the spittlebug. By incorporating the driving factors influencing the species life-history at the individual level, these models enable a physiologically-based (i.e. mechanistic) representation of population processes relevant for pest control like diapause termination, transitions between juvenile stages, and adult emergence^13–15^. Furthermore, phenological models can be straightforwardly implemented in decision support tools allowing easy access and use of information by managers and decision-makers^13,16^.

Several studies have explored the phenological dynamics of *P. spumarius* through monitoring data and experiments conducted under field or semi-field conditions. These investigations typically focus on estimating the duration of the development stages and their correlation with the observed temperatures^7,8,10,18–21^. Some studies have determined lower temperature thresholds for *P. spumarius* to describe stage durations based on the accumulation of degree-days (DD)^10,20^, while others have estimated linear DD functions to predict the spittlebug phenology^18,19,21^. However, there is still a lake of knowledge about non-linear stage-specific temperature-based development rate functions. Moreover, available studies often overlook the egg diapause process or assume diapause termination at an arbitrary point in time (e.g. January, 1st), after which the accumulation of heat is quantified. However, the diapause termination is a key process for the correct prevision of the phenology because it may significantly impact the timing of egg hatching and nymphal development. Recently, Lago et al.^21^ attempted to address this issue by testing different diapause termination dates fitting a phenological model to field data from the Iberian Peninsula. Despite this attempt, a physiologically-based function describing egg diapause termination in *P. spumarius* is still lacking, and the available knowledge regarding this process and its drivers remains limited.

The development of mechanistic phenological models requires the estimation of non-linear development rate functions at individual level. However, the life history of *P. spumarius* poses a challenge for establishing individual-based experiments due to the difficulty of maintaining individualised breeding of the preimaginal stages, making more convenient the development of cohort-based experiments. Although there are reliable demographic methods for the analysis of cohort studies^22–24^, their application can be complex and may impose specific assumptions that are sometimes incompatible with the obtained dataset. These limitations are particularly evident in experiments conducted with field populations, where there is limited control of the physiological age of individuals characterising the cohorts at the beginning of the experiment. In such cases, accurately estimating development rate functions becomes significantly challenging, as the observed distribution of stage durations can be attributed to both the natural intrinsic differences in developmental rates and to the distribution of the physiological age of the insects at the experiment’s onset. To overcome such barriers, researchers have proposed the use of mechanistic non-linear simulation models for the estimation of life history traits (i.e. stage-specific development functions) from cohort-based stage-frequency data^25,26^. This is a straightforward, flexible, and accurate approach that allows for incorporating the effects of forcing variables (e.g. temperature, photoperiod), considering the chronological or physiological age of the cohort in the model, and including the stochastic components to simulate individual variability in the phenology^13,15^.

In this paper we propose a physiologically-based simulation model to estimate the diapause termination process of eggs and the phenology of preimaginal stages of *P. spumarius* at high spatial resolution. Development rate functions were estimated from a cohort-based laboratory experiment. The model has been calibrated using field data collected in monitoring studies performed in olive groves in Liguria (Northern Italy) and Apulia (Southern Italy)^10,27^. The performance of the model was evaluated using field data collected in olive and cherry orchards in the Apulia region. The model provides useful information for the design and implementation of survey programs and Integrated Pest Management (IPM) strategies to control *P. spumarius* populations and support the management of *Xf* outbreaks in Europe.

## Results

### Development rate functions and initial distribution of physiological age of eggs

**Figure 1:**
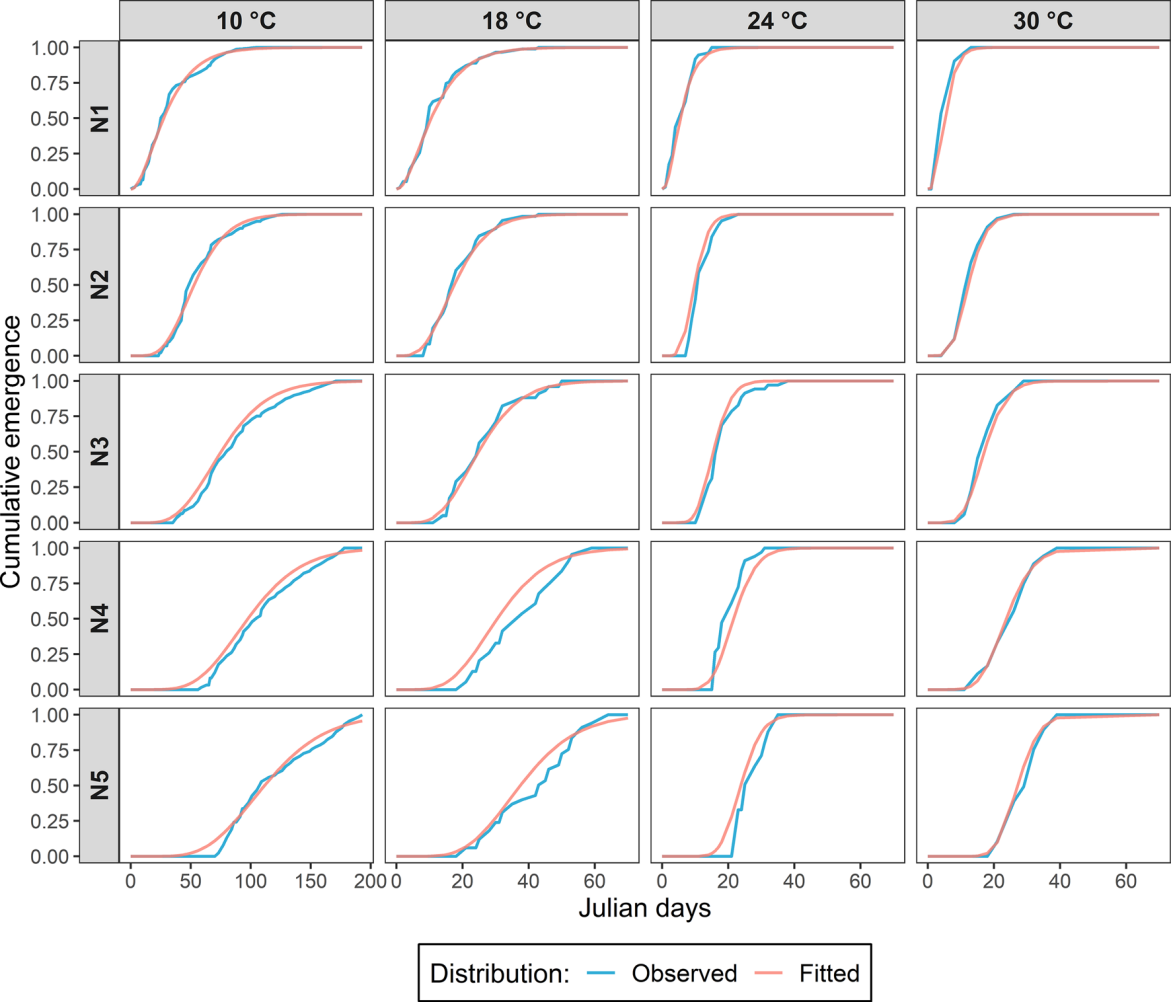
Comparison between the observed cumulative emergence curves of *P. spumarius* for each nymphal stage (rows) at each experimental temperature (columns) and the emergence cumulated curves obtained with the approximating Gamma distribution (fitted distribution). For sake of readability of the graphs, the x-axis scales of graphs referred to the temperature of 10 °C (0–200 Julian days) are different for the other graphs related to 18, 24 and 30 °C (0–70 Julian days).

**Figure 2:**
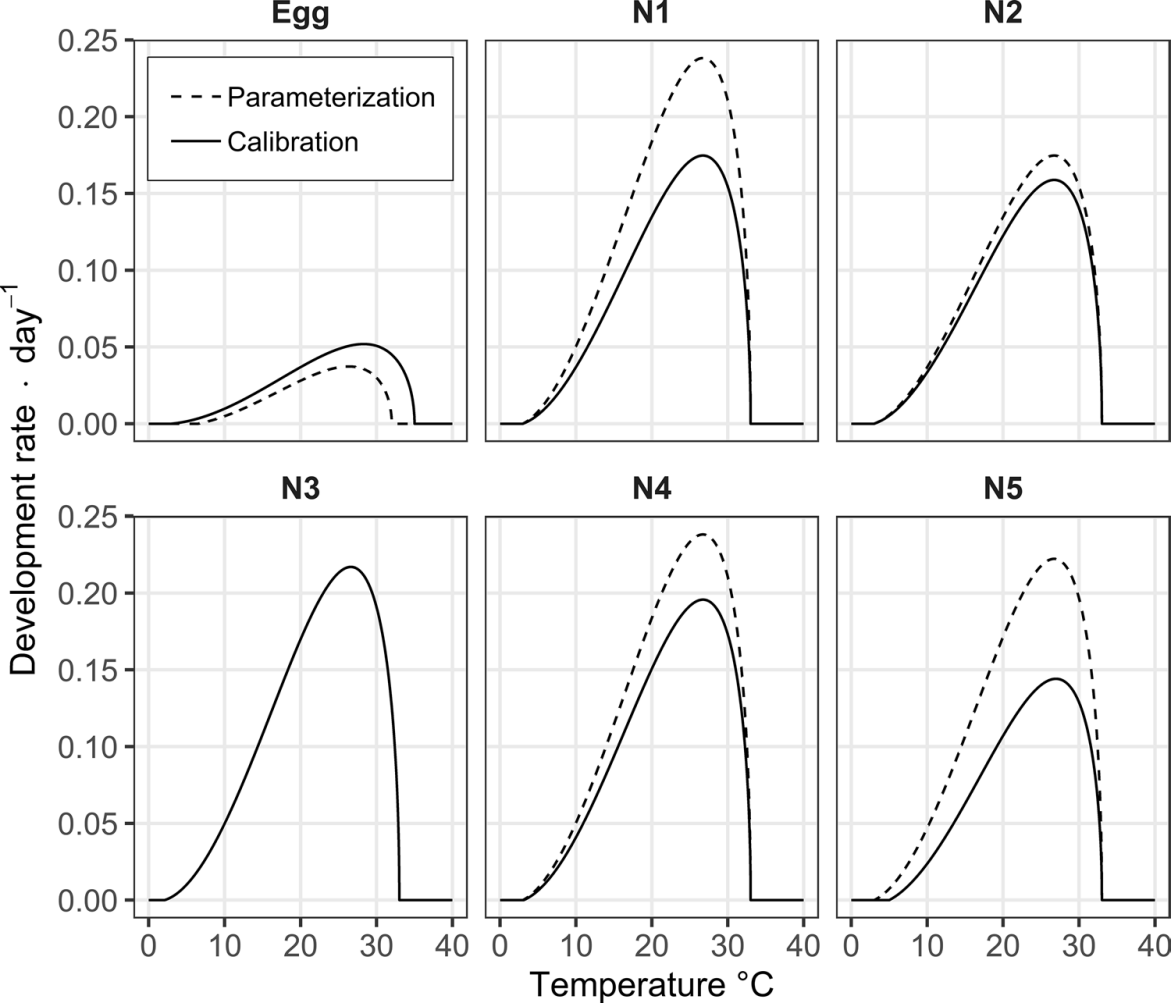
Temperature-based development rate functions of post-diapausing eggs and the five nymphal stages (N1–N5 in the charts) of *P. spumarius*, defined by the Brière function. The results of the parameterization procedure, based on climatic chamber experiments, are shown in dotted lines. The results of the calibration procedure, based on field data, are shown as continuous lines.

The emergence curves of preimaginal stages, obtained approximating the results of the climatic chamber experiments with a Gamma distribution, are represented in Fig. 1. Based on these curves, we estimated that the initial distribution of physiological age of eggs of *P. spumarius* was a Beta distribution with *α* = 5.67 and *β* = 1.05 (parameters obtained with the Kolmogorov minimization procedure). Therefore, 90% of the eggs were estimated to have a physiological age between 0.58 and 0.99 (i.e. they have completed between 58 and 99% of their development) at the beginning of the climatic chamber experiment, indicating an advanced embryonic development. The development rate functions of each stage are represented in Fig. 2 (dotted lines). The smallest development rate was observed for the post-diapausing eggs, presenting a maximum development rate equal to 0.0373 day^−1^ at 26.3 °C. The estimated lower and upper temperature thresholds for the development of post-diapausing egg were 6.5 and 32.0 °C, respectively. For the five nymphal instars, the estimates of lower and upper temperature thresholds and the optimum development temperature were quite similar, being approximately 3.0, 33.0 and 26.7 °C, respectively. The only exception is the third stage instar, presenting a lower temperature threshold of 2.1 °C and the optimal development temperature at 26.6 °C (Supplementary Table S1). The nymphal stages exhibit varying maximum development rates, with the 1st and 4th instars displaying the highest rate (0.2382 day^−1^), while the second instar demonstrates the lowest rate (0.1747 day^−1^).

### Model calibration

The developmental rate functions estimated during the parameterization procedure slightly changed after the calibration procedure (continuous line in Fig. 2; Supplementary Table S1). The optimized lower temperature threshold (3.0 °C) of the post-diapausing egg stage decreased by 3.5 °C compared to the former value (i.e. 6.5 °C), while the upper threshold and the optimum development temperature increased to 35.0 and 28.3 °C, respectively. The lower temperature thresholds of the 4th and 5th nymphal instars also increased compared to the former estimations, with the later instar presenting a more intense increase of 2.0 °C. The optimum temperatures remained similar between the five nymphal instars, ranging from 26.6 to 27.0 °C. No changes were needed in the estimations of upper temperature thresholds of nymphs. The maximum development rate of post-diapausing eggs increased to 0.0519 day^−1^, but remained the smallest value compared to the other insect stages. Except for the 3rd instar, whose rate estimate remains unchanged (0.2177 day^−1^), all nymphal stages presented a decreased maximum development rate compared to the estimated values in the parameterization procedure. The 5th instar presented the lowest estimates of maximum development rate (0.1441 day^−1^).

**Figure 3:**
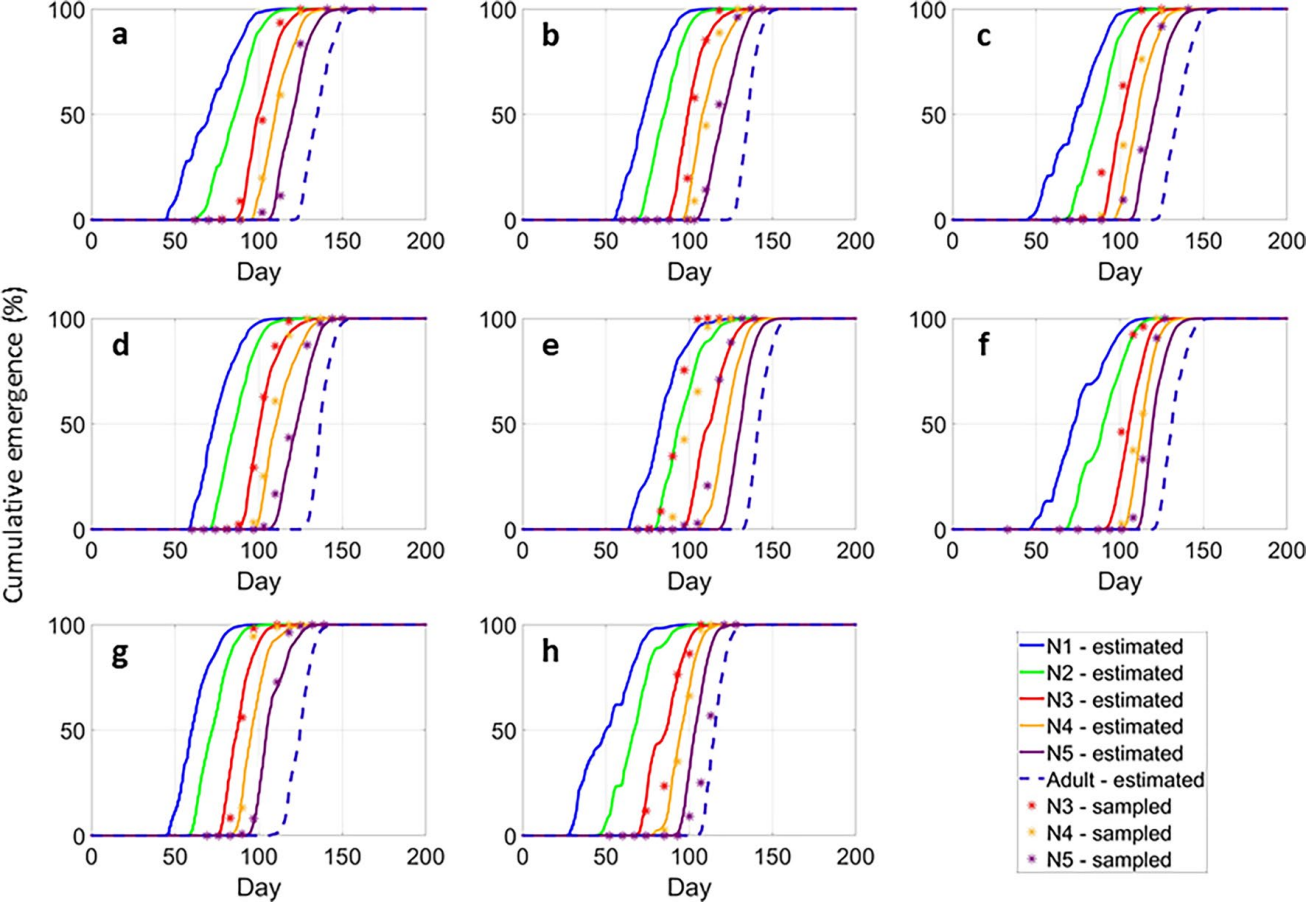
Results of the model calibration procedure. The sampled (asterisks) and estimated (lines) cumulative emergences (%) of *P. spumarius* stages considering the field experiments performed at (**a**) Arnasco in 2016 and (**b**) in 2017, (**c**) at Finale in 2016 and (**d**) 2017, (**e**) at Locorotondo in 2017 and (**f**) 2018, (**g**) at Valenzano in 2017 and (**h**) 2018. N1–N5 correspond to the 1st–5th nymphal instars, respectively.

During the calibration procedure, the temperature threshold ${T}_{e}$ and cumulative degree days ${DD}_{e}$ for termination of egg diapause were estimated at 6.5 °C and 120 DD, respectively. We estimated the conditions for diapause termination by focusing solely on the emergence curves of the 3rd, 4th, and 5th nymphal instars (Fig. 3). The estimated emergence curves of the 1st and the 2nd instars were unsatisfactory, exhibiting an anticipation of the beginning of the emergences compared to the field data in most cases. This discrepancy could be attributed to the challenges in obtaining accurate field data for the 1st and 2nd nymphal instars and to the uncertainties associated to the diapause termination process that can significantly impact the precision of the estimated phenology of these early stages. The observed emergence curve of the adult stage was not considered in our model since only the first adult appearances were used for the parameterization and calibration of the development rate functions. This approach was necessary because the shift in habitat occupation by adults after emergence demands distinct methodologies for surveying this stage.

### Model evaluation

**Figure 4:**
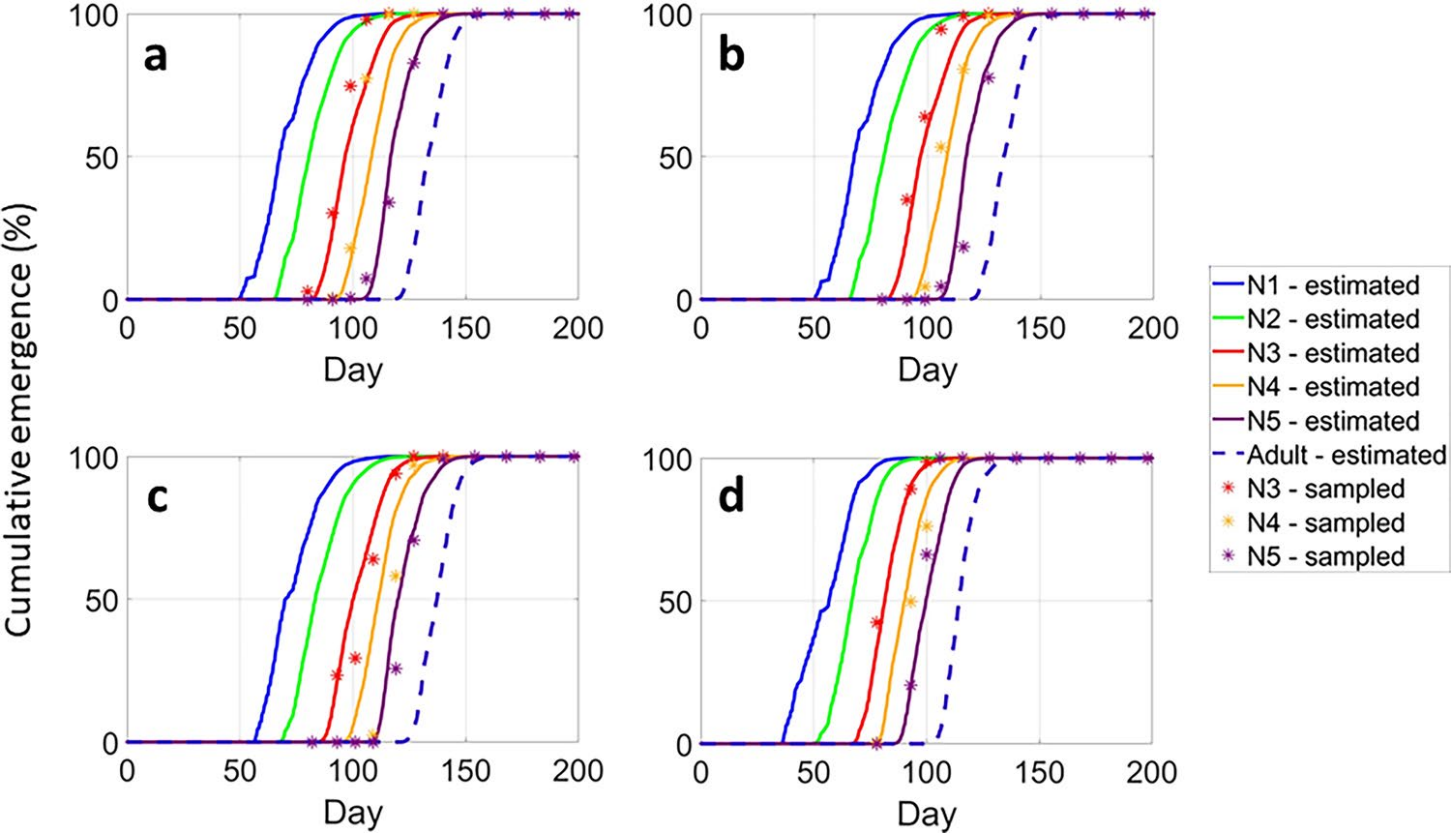
Results of the model evaluation procedure: comparison of the sampled (asterisks) and the estimated (lines) cumulative emergences (%) of *P. spumarius* stages in (**a**) a cherry grove in Castellana Grotte, and (**b**) in olive groves in Castellana Grotte, (**c**) Martina Franca, and (**d**) Surbo, in Apulia region (Southern Italy) in 2019. N1–N5 correspond to the 1st–5th nymphal instars, respectively.

The model exhibited a strong fit to the sampling data for the 3rd, 4th, and 5th nymphal instars, particularly during the initial half of the cumulative emergencies, precisely capturing the onset of stage emergence (Fig. 4). The observed emergence curve of the adult stage was not considered in our model for the same reason mentioned in the last section, although the simulated adult emergence curve is shown.

## Discussion

The diapause process of *P. spumarius* remains relatively underexplored in the literature. The few available studies are often influenced by the environmental and climatic conditions in which they were conducted and the specific traits of the spittlebug populations under investigation, lacking of generalization. For instance, Lago et al.^21^ proposed that the eggs leave diapause on December 1st, by fitting a model of development of post-diapausing eggs to field data from the Iberian Peninsula, while Chmiel and Wilson^18^ assumed that this process occurs in January 1st, based on field data of populations from the Northern United States. Our study is the first attempt to build a mechanistic approach describing the diapause termination in *P. spumarius* in terms of accumulation of heat energy by the diapausing eggs, providing a general model applicable to different climatic contexts. We estimated that the termination of eggs diapause occurs after accumulating 120 DD above a lower temperature threshold of 6.5 °C, starting from the 1st of January, this means, for example, that eggs typically start leaving diapause after mid-February in the Italian locations investigated in this paper. In our model, we rely on the simplified assumptions that only thermal accumulation triggers egg diapause termination, although the contribution of cold temperatures and photoperiod are reported as factors that influence the duration of diapause^7–9,17,28^. However, the possibility of including the cold temperature in our model requires the availability of a rate function describing the relationship between cold temperature and diapause duration, not yet explored in literature.

The estimated initial physiological age distribution of eggs is in line with the results of Weaver and King^7^ and Witsack^8^. They suggested that part of the embryonic development of *P. spumarius* occurs during fall. Consequently, first egg hatchings are expected to be observed shortly after diapause termination, with temperatures above the lower threshold for egg development. The range of the estimated distribution of physiological ages of eggs (varying from 0.58 to 0.99) might be partially in contrasts with the study of Witsack^8^ on the embryonic development of *P. spumarius* during the onset of diapause. This author observed that eggs entered diapause in a specific step of embryogenesis, without providing information on the physiological age of this transition neither an associated estimation of inter-individual physiological ages. The wide range of physiological age obtained from the optimization procedure adopted for our model estimation could be possibly an outcome of uncontrolled variables intervening in the experimental process together with inter-individual variability in the physiological age at the onset of diapause.

The lower temperature threshold of post-diapausing eggs (3.0 °C) is lower than those defined by Chmiel and Wilson^18^ of 6.5 °C, and by Lago et al.^21^ of 9.2 °C. In the latter study, the authors estimated an optimum temperature for the post-diapausing eggs of 23.4 °C, substantially lower than our estimate (28.3 °C), although the upper development thresholds were similar (35 °C). However, the experimental design followed by Lago et al.^21^ and the functional form of the development rate function is different from the non-linear function used in our model, these two elements could partially account for the different results obtained. Chmiel and Wilson^18^ estimated a lower temperature threshold for nymphs similar to the one we estimated (3.0 °C), but lower than the 5.0 °C assumed by Halkka and Halkka^29^. Our estimate of the upper development temperature threshold of 33.0 °C for the nymphs is much higher than the results of the study of Chmiel and Wilson^18^. These authors estimated an upper threshold equal to 26.7 °C, a value closer to the optimal development rate temperatures estimated in our study. The possibility that differences in the development rate functions of the egg and the nymphal stages we obtained compared to the values found by other authors can be related to the different adaptation to local climatic conditions (especially during late winter and spring, to which the spittlebug populations are exposed) remains a factor to be investigated.

Control strategies targeting the nymphal population are important to reduce the further emergence of adults in the field, which are effectively responsible for transmitting *Xf* to the crops^1,4,10^. The main management strategies of the spittlebug nymphs involve ground cover removal (i.e. through soil tillage of the crop rows and edges, and mechanical or chemical weed control), and sprays of chemical or biological (still little explored) insecticides^1,30,31^. The phenological curves estimated for the 3rd–5th nymphal instars by our phenological model presented a satisfactory fit to the field populations in location where the model has been tested in Italy. This suggests that our phenological model is appropriate for the estimation of the emergence of the three late nymphal stages, which are the key stages to target monitoring actions and control measures against *P. spumarius*. According to Bodino et al.^10^, the peak of emergence of the 4th instar nymphs represents a crucial moment for maximizing the efficacy of management strategies targeting the nymphal population, since it coincides with the peak of the nymphal abundance, when no more nymphs eclose, and no adults are yet present. These phenological dynamics can also be observed in the population surveys conducted by Chmiel and Wilson^18^, Zajac et al.^19^, Antonatos et al.^32^, and Beal et al.^20^, which were performed in different agroecological landscapes contexts and climatic conditions.

Even though our model is able to predict the emergence curve of *P. spumarius* adults, we could not evaluate the simulated emergence patterns due to the lack of real data. The adults present a greater dispersal ability than the nymphal stages and usually leave the herbaceous cover once they emerge and move onto shrubs and trees^4,28^. This shift in habitat occupation demands distinct methodologies for surveying this stage, making it challenging to gather field data that captures the cumulative emergence necessary for the evaluation of the model’s predictions. Nevertheless, the model provides valuable biological information about adult emergence that contributes significantly to the understanding of the species phenology and the integration of the model into epidemiological models.

The estimated phenological model emerges as a useful rational decision support tool guiding growers and decision-makers scheduling monitoring and control actions against the late nymphal stages of *P. spumarius*. The model provides accurate information for the identification of appropriate phenological timing for interventions, as it can simulate local phenology based on the weather temporal trends, thus enhancing management precision. Furthermore, it can be used to generate maps at different spatial resolutions to manage actions at the level of productive patches, incorporating the role of spatial variability in the vector’s phenological progression. The model can be easily adapted to be used in other regions through further parameter calibration using reliable field datasets from the contexts of interest. It can also be implemented into *Xf* epidemiological model frameworks (e.g. Gilioli et al.^33^), in order to enhance the realism of the representation of the vector dynamics. In this way, such complex models can guide researchers and decision-makers to investigate optimal management strategies under the IPM context to prevent and mitigate the spread of the *Xf* epidemics. In addition, they can also provide important insights, improve the knowledge about this complex epidemiological system, and highlight the research topics and gaps that need to be approached. In the context of our study, there are still important limitations in the understanding of the biological processes involved in the diapause dynamics of *P. spumarius* eggs that are important to be investigated in more detail to allow a more realistic estimation of the diapause termination and egg hatching moments. An effort should be made to develop optimized monitoring designs for the spittlebug populations to improve the precision of the surveys in the field^1^, which is a very important decision-making tool for an assertive implementation of control measures.

## Methods

### Phenological model

The phenological dynamics of *P. spumarius* are simulated through a stage-structured population model based on the system of Kolmogorov partial differential equations considering two dimensions: time *t* and physiological age *x* (for more details see Gilioli et al.^13,14^; Pasquali et al.^15,34^). We considered seven developmental stages (*i*), namely egg (*i* = 1), the five nymphal instars (*i* = 2–6), and the adult stage (*i* = 7). For the adult stage, only the flux of individuals transferred from the fifth nymphal instar was modelled. Phenological processes specific to the adult stage, such as parapause initiation and termination, as well as ovary maturation, are omitted from the model due to the absence of suitable data. The physiological age of an individual in the *i-*th stage, ${x}^{i}\in [0, 1]$, is defined as the proportion of individual development within its stage, in which ${x}^{i}=0$ is the age at the beginning of the *i-*th stage and ${x}^{i}=1$ is the age when the stage development is completed and the individual passes to the next stage $i+1$ ^35^. The stage-specific temperature-based development rate function, ${v}^{i}\left(T(t)\right)$, is modelled with the Brière function^36^

1 $$v^{i} \left( {T\left( t \right)} \right) = \left\{ {\begin{array}{*{20}c} {a^{i} T\left( t \right)\left( {T\left( t \right) - T_{\inf }^{i} } \right)\sqrt {T_{\sup }^{i} - T\left( t \right)} } & {T_{\inf }^{i} \le T\left( t \right) \le T_{\sup }^{i} } \\ {0 } & { T\left( t \right) < {T_{\inf }^{i} \space \text {or} \space T\left( t \right)} > T_{\sup }^{i} } \\ \end{array} } \right.$$

where ${a}^{i}$ is an empirical constant, $T(t)$ is the air temperature at time *t* and ${T}_{inf}^{i}$ and ${T}_{sup}^{i}$ are the lower and the upper development temperature thresholds, respectively.

The initial condition of the phenological model is defined as 100 diapausing eggs and zero individuals for the other stages on the 1st of January. We assumed an initial distribution of the physiological ages of eggs, due to the variability in the pre-diapausing development phase of eggs. The development process of eggs is stopped during diapause. We also assumed that the diapause ends when the eggs accumulate enough heat energy (*DD*_*e*_) described in degree days above a minimum temperature threshold (*T*_*e*_). The post-diapausing eggs continue to develop until eggs hatching (*x*^1^ = 1).

### Model parameterization

The parameters ${a}^{i}$, ${T}_{inf}^{i}$ and ${T}_{sup}^{i}$ of the temperature-dependent development rate functions for the preimaginal stages (Eq. 1), as well as the initial Beta distribution of physiological age of eggs, are estimated from a set of population dynamics of *P. spumarius* analysed in a climatic chamber at different constant temperature (D. Bosco and N. Bodino, personal information). These data are graphically represented in Fig. 1. The eggs used in the experiment were obtained from microcosms (cages) containing a couple of adults (male and female) used for oviposition and placed in open-air conditions in Turin (Northern Italy). Eggs were collected from September to the beginning of November and stored at the temperature of 4 ± 1 °C to slow down their development until the beginning of the experiments and to maximize their probability of survival^8^. In mid-February, the eggs were removed from storage at 4 °C, placed in climatic chambers at four constant temperatures (10, 18, 24, and 30 °C) and followed in their development until adult emergence or death. Nine replicates for each temperature were carried out. The number of eggs hatched and the number of individuals in each nymphal stage were recorded three times a week.

The cumulative emergence curves, representing the phenological dynamics of preimaginal stages of *P. spumarius* for each temperature, were approximated by Gamma distributions. Since there was no control of the oviposition date of the eggs collected in the microcosm, the physiological age of the eggs at the beginning of the experiment can vary considerably. Due to the characteristics of the climatic chamber experiment, we did not consider the diapausing phase in this step.

The Kolmogorov model has been used for simulating the emergence of both the preimaginal and the adult stages. The stage-specific development rates and temperature thresholds, as well as the initial physiological age distribution of eggs (based on the Beta distribution), have been estimated by fitting the experimental data with the Kolmogorov model through the following minimization procedure. We iteratively modified some of the model parameters (i.e. the stage-specific development curves and the initial physiological age distribution of eggs) to minimize the differences between the four observed phenological curves of the preimaginal stage and the estimated ones (function *fmincon,* MATLAB v. 9.14 R2023a). As stopping conditions, we set the threshold related to the difference in the parameter’s values between steps (step tolerance = 1.0e−6) and the difference in the error minimization (function tolerance = 1.0e−1).

### Model calibration

In this step, we calibrated the parameters of the development rate functions, initially estimated through experiments under controlled conditions as outlined in the preceding section, with data collected in field conditions. Furthermore, we estimated the conditions triggering the diapause termination.

We used data on preimaginal stages dynamics of *P. spumarius* collected in four olive orchards in Italy: at Arnasco and Finale in 2016 and 2017 (Liguria region, Northern Italy), at Locorotondo and Valenzano in 2017 and 2018 (Apulia region, Southern Italy)^10,27^. The data consists of weekly samplings of the spittlebug nymphal populations and first adults on the herbaceous vegetation in an olive grove, from early March until late May. Hourly temperature data are obtained from data loggers installed in each olive grove, exposed to similar conditions to those experienced by the spittlebugs present on the ground cover.

The calibration procedure was divided into two steps: (i) we calibrated the parameters ${a}^{i}$, ${T}_{inf}^{i}$ and ${T}_{sup}^{i}$ of the stage-specific temperature-dependent rate functions, then (ii) we estimated the most appropriate values for the minimum temperature threshold ${T}_{e}$ and the cumulative degree-days ${DD}_{e}$ for the diapause termination process. Based on previous studies^7,8,21^, we tested several combinations of values for the parameters ${T}_{e}$ (ranging from 3.0 to 10.0 °C) and ${DD}_{e}$ (from 30 to 130). For the simulations performed in the model calibration, the physiological age distribution of diapausing eggs was assumed equal to initial physiological age distribution estimated in the model parameterization.

### Model evaluation

This phase aims to evaluate the performance of the calibrated phenological model on new phenological dynamics data, not considered in the calibration procedure. We used data collected in the Apulia region during the monitoring survey in 2019 (Dongiovanni, unpublished data). We focus on three olive groves in Castellana Grotte, Martina Franca and Surbo, and one cherry grove in Castellana Grotte. The data consists of weekly samplings of preimaginal stages of *P. spumarius* on the herbaceous cover of the grove, from the second half of March until late May. The temperature data for model evaluation was obtained from the fifth generation European ReAnalysis (ERA5-Land) dataset, which reports estimations of hourly air temperatures at a 0.1° × 0.1° spatial resolution^37^.

### Supplementary Information

Supplementary Information.

## Data Availability

The datasets generated during and/or analysed during the current study are available from the corresponding author on reasonable request.

## Code availability

Simulations of the phenology of *Philaenus spumarius* have been performed using a custom code.
