# Supplementary material for: A model for predicting the phenology of Philaenus spumarius
Source: Sci Rep. 2024 Apr 7;14:8137. doi: 10.1038/s41598-024-58798-x (PMC10999437; doi:10.1038/s41598-024-58798-x)
Supplement: Supplementary file 1 — Supplementary Information. [file 41598_2024_58798_MOESM1_ESM.pdf]

Supplementary Information

A model for predicting the phenology of *Philaenus spumarius*

Gianni Gilioli[1*], Anna Simonetto[1], Igor Daniel Weber[1], Paola Gervasio[1], Giorgio Sperandio[1,2], Domenico Bosco[3], Nicola Bodino[3], Crescenza Dongiovanni[4], Michele Di Carolo[4], Vincenzo Cavalieri[5], Maria Saponari[5], Donato Boscia[5]

[1] University of Brescia, DICATAM, Via Branze 43, 25123, Brescia, Italy
[2] Marche Polytechnic University, D3A, Via Brecce Bianche 10, 60131, Ancona, Marche, Italy
[3] University of Turin, DISAFA, Largo Paolo Braccini, 10095 Grugliasco (TO), Italy
[4] Centro di Ricerca, Sperimentazione e Formazione in Agricoltura Basile Caramia, Locorotondo, Italy
[5] Consiglio Nazionale delle Ricerche, Istituto per la Protezione Sostenibile delle Piante, Sede Secondaria di Bari, Bari, Italy

[*]Email: gianni.gilioli@unibs.it

Table S1. Values of the parameters *a*, T_{inf} (minimum temperature threshold, °C) and T_{sup} (maximum temperature threshold, °C) of the temperature-dependent development rate functions based on Brière equation, of post-diapausing eggs and 1st–5th nymphal instars (N1–N5) of *Philaenus spumarius*, after the parameterization and calibration processes. The values of T_{opt} (optimum development temperature, °C) and r_{max} (maximum development rate, day^{-1}) were obtained from the estimated functions.

Stages	Parameterization					Calibrated				
	a	T_{inf}	T_{sup}	T_{opt}	r_{max}	*a*	T_{inf}	T_{sup}	T_{opt}	r_{max}
Egg	0.00003	6.5	32.0	26.3	0.0373	0.00003	3.0	35.0	28.3	0.0519
N1	0.00015	3.0	33.0	26.7	0.2382	0.00011	3.0	33.0	26.7	0.1747
N2	0.00011	3.0	33.0	26.7	0.1747	0.00010	3.0	33.0	26.7	0.1588
N3	0.00013	2.1	33.0	26.6	0.2177	0.00013	2.1	33.0	26.6	0.2177
N4	0.00015	3.0	33.0	26.7	0.2382	0.00012	3.1	33.0	26.7	0.1960
N5	0.00014	3.0	33.0	26.7	0.2224	0.00010	5.0	33.0	27.0	0.1441
